# Technologies for Pharmacogenomics: A Review

**DOI:** 10.3390/genes11121456

**Published:** 2020-12-04

**Authors:** Maaike van der Lee, Marjolein Kriek, Henk-Jan Guchelaar, Jesse J. Swen

**Affiliations:** 1 Department of Clinical Pharmacy and Toxicology, Leiden University Medical Center, 2333ZA Leiden, The Netherlands; m.vanderlee@lumc.nl (M.v.d.L.); h.j.guchelaar@lumc.nl (H.-J.G.); 2 Leiden Network of Personalized Therapeutics, 2333ZA Leiden, The Netherlands; 3 Department of Clinical Genetics, Leiden University Medical Center, 2333ZA Leiden, The Netherlands; m.kriek@lumc.nl

**Keywords:** pharmacogenomics, genotype, phenotype, next generation sequencing, long-read sequencing

## Abstract

The continuous development of new genotyping technologies requires awareness of their potential advantages and limitations concerning utility for pharmacogenomics (PGx). In this review, we provide an overview of technologies that can be applied in PGx research and clinical practice. Most commonly used are single nucleotide variant (SNV) panels which contain a pre-selected panel of genetic variants. SNV panels offer a short turnaround time and straightforward interpretation, making them suitable for clinical practice. However, they are limited in their ability to assess rare and structural variants. Next-generation sequencing (NGS) and long-read sequencing are promising technologies for the field of PGx research. Both NGS and long-read sequencing often provide more data and more options with regard to deciphering structural and rare variants compared to SNV panels—in particular, in regard to the number of variants that can be identified, as well as the option for haplotype phasing. Nonetheless, while useful for research, not all sequencing data can be applied to clinical practice yet. Ultimately, selecting the right technology is not a matter of fact but a matter of choosing the right technique for the right problem.

## 1. Introduction

The field of pharmacogenomics (PGx) is developing rapidly. The first PGx dose recommendations for antidepressant and psychiatric drugs were published in 2001, even before the first human genome was sequenced [1]. An increase in available evidence and the ambition to implement PGx in clinical practice has led to the need for more comprehensive dosing guidelines and genotyping strategies. In 2005, the Dutch Pharmacogenetics Working Group (DPWG) was formed to develop evidence-based PGx guidelines [2]. In 2011, the Clinical Pharmacogenomics Implementation Consortium (CPIC) was founded [3]. Currently, CPIC and the DPWG combined have issued PGx dose recommendations covering more than 50 drugs and 21 genes (Table 1) [4,5]. 

In the last 20 years, there has not only been much progress in development of PGx guidelines, there has also been a significant technological advancement and rise of new technologies for assessing genetic variants. At the time of development of the first PGx guideline, only Sanger-based sequencing techniques and SNV (single nucleotide variant) arrays were available as methods for variant identification. To date, SNV panel testing remains the most commonly used technology in clinical practice. However, while it is efficient and comes at low costs, SNV panels cannot detect all important genetic variation such as rare and structural variants. Currently, multiple high throughput whole genome sequencing techniques are available, yielding an abundance of genetic information at a fraction of the costs of 20 years ago [6,7,8,9,10]. Nevertheless, these approaches are not yet routinely used in other clinical fields, despite their potential [11,12]. 

In this paper, we review the application of genotyping technologies for PGx. We first discuss the use of SNV panels, which are the most commonly used approach for clinical PGx. Next, we discuss the potential of next generation sequencing (NGS) and long-read sequencing and their current use in PGx. We review the challenges in PGx, both for clinical as well as research purposes, and the way PGx technologies can help in solving them. We mainly focus on germline variants and their role in PGx. Nonetheless, the outlined principles hold true for somatic mutations [13,14,15]. Additionally, implementation and adoption of PGx in clinical practice is outside the scope of this review and has been extensively discussed in previous publications [16,17,18].

## 2. SNV Panels: Current Clinical Practice

SNV panel testing is the most commonly used technology in PGx practice, either through commercially available micro-array platforms or with custom arrays. The arrays typically contain a preselected set of SNVs, which, depending on the array and platform, can range from a few variants in a single gene to thousands of variants genome wide. Commercially available PGx arrays typically contain variants that are linked to drug response in PGx guidelines or on PharmGKB [19]. The evidence underlying the selected variants can vary, from small arrays containing only the most strongly associated variants, to very large arrays containing all variants potentially or theoretically associated with drug response—for example, including all known drug-related genes. Almost all available arrays use PCR, sequencing by synthesis and nanospheres or beads, combined with a form of fluorescence or chemiluminescence detection to identify which variant is present at the site of interest [20,21,22]. Another technology is the use of mass spectrometry, relying on differences in mass between wildtype and mutant nucleotides [23]. Detailed descriptions of these techniques have been described previously [3,24,25,26,27,28]. The pre-selection of variants and the relatively low amount of data to process allow for a quick result at low costs (Table 2).

### 2.1. Commercial Arrays

There are many arrays available that can be used for PGx; a full overview of these arrays is beyond the scope of this review. Two of the smaller commercial arrays are the VeraCode ADME core panel (Illumina Inc. San Diego, CA, USA) and the VeriDose core panel (Agena BioScience, San Diego, CA, USA). The VeraCode consists of 184 variants in 34 pharmacogenes [20] and the Veridose contains 68 variants in 20 genes and 5 copy number targets for CYP2D6 [30]. The ADME core variant list is based on an expert gene panel and contains the most biologically relevant variants within these genes [20,31]. For the VeriDose, genes with a known clinical impact and their common clinically actionable variants are selected. Additionally, it is possible to expand the panel if so desired [30]. Both panels provide sufficient coverage for clinical PGx by covering the most common variants in actionable pharmacogenes. More extensive panels are the pharmacofocus with (2000 variants in 150 genes including CNVs (Copy Number Variants) (ThermoFisher Scientific, Waltham, MA, USA) [32] and the pharmacoscan with 4627 variants in 1191 genes (ThermoFisher Scientific) [22]. The latter contain nearly all variants from the DMET and Illumina ADME core panel, in addition to all genes and variants with clinical annotations in CPIC and PharmGKB (Pharmacogenomics knowledge base), HLA genes and sample ID and tracking markers. These types of arrays are widely used in clinical PGx implementation studies. For example, the DMET array is used in the PG4KDS study from St. Jude’s children’s research hospital [3] and the VeraCode ADME core panel is used in the PREDICT study [33]. Both these studies use only a subset of the variants available on the panel for the clinical implementation part of the studies [3,33]. Only the variants in the genes of interest and the variants with sufficient data are reported for clinical practice. The remainder of the genetic data is, with informed consent permission, stored and can be used for research purposes or for later clinical use. For a full overview of studies using PGx panel approaches, we refer to previous publications providing such an overview [11,34].

### 2.2. Custom Arrays

Several of the commercial arrays contain a high number of variants, making a fast turnaround time and interpretation challenging. Additionally, these arrays will include variants which may not be of direct interest in a clinical setting as panels often include all known PGx variants regardless of the level of evidence supporting their clinical utility. This has driven many institutions to develop their own custom clinical array with a more focused set of clinically actionable variants. Typically, these custom arrays are performed using single gene-based testing covering only the variants with direct clinical applicability yet limiting broad applicability. To be able to test a broad set of variants while maintaining a rapid turnaround time, companies have developed customizable arrays. One example frequently used for clinical PGx is the OpenArray (Thermofisher scientific). This array can detect between 12 and 240 variants using standard TaqMan technology [35]. Selected TaqMan assays are spotted on a chip based on the clients need. Each chip contains wells for the patient samples; in each of these wells, through-holes are present which contain the assays desired. This format allows for a thorough characterization of a few genes as well as for a broader approach focused on a panel of common variants in multiple genes. This array has been used in clinical implementation studies as well. For example, in the INGENIOUS study it was used to interrogate a panel of 43 variants in 14 genes [36]. A similar approach was used by the Ubiquitous pharmacogenomics (U-PGx) consortium. The U-PGx consortium’s initiated the PREPARE study aimed at collecting evidence of the clinical utility of a pre-emptive PGx panel consisting of 58 SNVs in 14 pharmacogenes [34]. The panel covers the most common variants in all actionable genes included in the DPWG guidelines [37] and is analyzed with KASP technology using the SNPline (LGC) [38].

### 2.3. Array Developments

The above-mentioned commercial and custom arrays are developed specifically for PGx. However, there are also multiple arrays with genome-wide coverage available. An added benefit of genome wide arrays is that they also allow for GWAS (Genome Wide Association Study) analysis in addition to providing PGx information. Examples of such arrays are the Illumina GSA (Global Screening Array) which contains over 600,000 SNVs genome-wide, including 17,750 PGx markers [39] and the Axiom arrays (Thermofisher scientific), which contain genome-wide coverage specifically for a certain population [40]. Nonetheless, these arrays often miss dense coverage of PGx regions and not all critical SNVs are available on the array. For example, in the case of the GSA v3.0, the SNVs for CYP2D6*4 and CYP2C19*9 and target CNV testing are not included. This is particularly concerning for the *CYP2D6* gene. The CYP2D6 enzyme is responsible for 25–30% of commonly prescribed drugs, making sufficient coverage on variants on the *CYP2D6* gene clinical important [41]. The CYP2D6*4 allele is the most frequent null-allele in Caucasians, with the key SNV (rs3892097; NC_000022.11:g.42128945C>T) occurring in 19% of the European (non-Finnish) population [42,43]. Additionally, the GSA v3.0 does not contain probes for direct CNV detection.

## 3. Next Generation Sequencing

### 3.1. Next Generation Sequencing Technologies

NGS technologies are not yet routinely applied in clinical PGx. However, they are often used in PGx research and disease genetics. While SNV panels only cover a limited set of selected variants, sequencing data cover the full exome or genome. Technical details of NGS technologies have been extensively reviewed elsewhere [44,45,46]. In short, NGS technologies are capable of sequencing reads of 100–200 bp in a high throughput manner, allowing for the sequencing of a full genome in a matter of hours. These reads are aligned to the reference genome and variants are identified based on deviations from the reference.

NGS applications can be roughly categorized into three approaches. First, whole exome sequencing (WES) focusing on sequencing the coding regions of the genome, covering approximately 1–2% of the entire genome. Secondly, whole genome sequencing (WGS) which is aimed at sequencing the entire genome, both coding and non-coding regions. Lastly, targeted sequencing of a region or panel of genes of interest [44,45,46]. While NGS can be performed at relatively low costs, the large amount of data makes processing more challenging (Table 2).

### 3.2. Use of NGS for Pharmacogenomics

While NGS has become the standard for clinical diagnostics and in research, it is yet to be widely adopted for clinical PGx. Nonetheless, in a research setting NGS has been used for PGx for several years. Multiple studies have been conducted investigating the accuracy of NGS technologies in PGx as well as the applicability of an NGS sequencing panel or of the repurposing of clinical NGS data for PGx [9,10,47,48,49,50,51]. Yang et al. performed a three-way analysis with the DMET, WES and WGS, to investigate the concordance between PGx genotyping calls based on these different technologies. They showed a 94% concordance between the DMET and WES, and a 96% concordance between the DMET and WGS [47]. Similar results were reported by other groups, all of which report the superior results obtained from sequencing compared to orthogonal testing [49,50,51,52,53]. The difference in concordance between WES and the DMET array (94%), and WGS and the DMET array (96%) can be explained by the genomic coverage of each approach. WES only covers the exons and can therefore, by definition, not cover all relevant variants if they are located in the intronic or intergenic regions. WGS, on the other hand, also covers intronic regions leading to an expanded coverage. Nonetheless, intronic variants are of clinical importance in PGx. For example, one of the key CYP2C19*17 variants (rs12248560; NC_000010.11:g.94761900C>T) is located upstream of the *CYP2C19* locus. Other examples are CYP3A5*3 and *5 as well as CYP2D6*4 and *41 [54]. A targeted sequencing approach can combine the lower costs of WES with the advances of WGS data. This type of panel only captures genes of interest, both intronic and exonic regions. This results in lower costs while maintaining the accuracy and abundance of data of WGS. One such approach is the PGRNseq panel [52]. This panel is based on full-gene sequencing of a panel of 84 pharmacogenes using NGS, it has also been used in clinical implementation studies showing promising results [49,55]. Another approach is the PGxSeq panel described by Gulilat et al., which covers 100 pharmacogenes [56].

### 3.3. Repurposing of Clinical Genetics Data

The abovementioned approaches are aimed at generating novel sequencing data with the goal of providing PGx results, whether it be panel, WES or WGS-based. However, in clinical diagnostics, the use of NGS is already standard practice in many centers leading to vast amounts of sequencing data. Several groups have investigated the feasibility of repurposing these data for PGx, by extracting a panel of evidence-based PGx variants from the data and translating this into a clinically applicable result [9,10]. The same type of analysis is performed for large populations studies, such as the Estonian biobank [8], the University of Colorado [57], SWEDEGENE [7] and the AllofUs initiative [58,59]. Unfortunately, the utility of the repurposing of data is dependent on the capture panel used in the original sequencing. This is particularly a problem in the use of WES data, as mentioned above, several important PGx variants are located in intronic regions which are not included in WES capture kits [54]. Nonetheless, even with these limitations, high percentages (>85%) of individuals with actionable phenotypes are identified [9,10,60], but one should bear in mind that important variants are missing.

## 4. Long-Read Sequencing

### 4.1. Long-Read Sequencing

Long-read sequencing technologies have emerged in the playing field and are slowly gaining ground over the short-read approaches in the field of research [46,61]. Both Pacific Bioscience (PacBio) technology as well as Oxford Nanopore Technologies (ONT) are becoming an integrated part of genetic approaches [12]. PacBio uses SMRT (single molecule real-time)-sequencing to be able to sequence reads up to 45 kB. SMRT cells make use of microwells, each of which contains one single strand of DNA which is then sequenced by assembly and recorded in real time [62]. Oxford Nanopore Technologies uses nanopores through which the DNA strand is pulled, the disruption in the current is specific to a codon, allowing for the full assembly of the DNA sequence [63]. By correcting for the randomly distributed errors in single cell sequencing, the consensus reads can obtain very high accuracy [12,62,64]. While an abundance of data can be generated by long-read sequencing, the processing is significantly more intensive compared to SNV panels and NGS (Table 2).

### 4.2. Long-Read Sequencing for PGx

Long-read sequencing has been shown to be capable of solving complex loci genome wide [12,64]. Current disease diagnostics already use long-read sequencing for complex genetic diseases such as the *ATXN10* repeats in Parkinson’s disease [65] and tandem repeats in the *FMR1* gene associated with Fragile X syndrome [66]. Nonetheless, only a few long-read sequencing studies for pharmacogenes have been conducted. The most thoroughly investigated complex locus in PGx is the *CYP2D6* gene, which contains both SNVs and SVs. (Structural Variants) It has been shown that with long-read sequencing, the *CYP2D6* locus (~6.6 kbp) can be sequenced in one full read and be fully resolved into phased haplotypes, including structural variants [67,68]. The same has been observed for the notoriously complex HLA genes [12,64]. Long-read sequencing in PGx is currently, to our knowledge, limited to single gene studies and no large-scale studies applying long-read sequencing for clinical PGx to a panel of genes have been conducted.

## 5. Challenges

### 5.1. Drug Metabolizer Phenotype Inference

Prior to application in clinical practice, SNV data are translated into predicted drug metabolizer phenotypes. Many of the arrays mentioned above are developed based on the SNVs which are present in the genotype nomenclature. For CYP enzymes, haplotypes are named with the star (*) nomenclature [42,69]. All variants making up a *-allele are described by the Pharmacogene variation Consortium (PharmVar, https://www.pharmvar.org). The combination of the two *-alleles present in an individual is subsequently translated into a predicted drug metabolizer phenotype. For most pharmacogenes, there are four metabolizer phenotypes: normal metabolizers (NMs) displaying full protein function, intermediate metabolizers (IMs) associated with decreased protein function, poor metabolizers (PMs) indicating absence of protein function and ultra-rapid metabolizers (UMs), which are associated with increased enzyme function.

Depending on the number of variants and the presence of the variants in translation guidelines, the interpretation is relatively straightforward. However, if there are many variants leading to *-haplotypes of unknown function present on the array, the interpretation is challenging. Furthermore, PharmVar describes the *-haplotypes extensively, defining large numbers haplotypes and sub haplotypes which are constantly increasing. In theory, all variants defined by PharmVar at a certain point in time could be included on an SNV panel. Nonetheless, with over 2000 variants known in CYP2D6 alone, this would quickly grow to a very large panel which is difficult to apply and interpret in clinical practice [42]. As there is no standardization in regard to the variants which need to be tested in a clinical setting, every array contains its own set of variants. This can lead to differences in regard to assigned haplotypes when testing the same DNA in different laboratories [49]. For example, results from the pharmacoscan (4627 variants) will be much more extensive and detailed compared to the VeriDose Core panel (68 variants) [22,30]. The pharmacoscan analysis might also result in more variants and haplotypes of unknown effect while, on the other hand, the VeriDose core panel might miss variants with a known effect.

The use of sequencing data enables the inclusion of almost every known variant in the *-haplotype assignments and subsequently into predicted phenotypes. However, with this abundance of variant data comes an increased difficulty in haplotype assignments. Several tools have been developed to assign *-allele haplotypes based on sequencing data, incorporating all variants in the assignment. Yet, as Caspar et al. have shown, these tools are not performing perfectly with errors in the assigned haplotypes compared to the consensus genotype from GeT-RM [60]. An error is here defined as a haplotype assignment which differs from the consensus. The best performing tool was Aldy [70] with 2 out of 21 errors. Astrolab [71] and Stargazer [72] both performed worse with 9 and 10 errors, respectively. Similar results were reported by Twesigomwe et al. in a CYP2D6 specific study [73]. All tools use the variants and *-allele translation in PharmVar as their database. However, PharmVar is updated continuously leading to potential differences in assignments if not every tool is updated at the same time. Additionally, depending on the data on which the tools are trained and tested, they might be more sensitive for specific variants and alleles [60]. Even the most extensive tools in regard to *-haplotype calling might not be suitable for clinical practice as they contain variants of which the effect is unknown. Furthermore, for clinical implementation only, the variants of known functional effect are relevant, resulting in a need for translation tools focused on clinical implementation only. However, selecting which variants are of direct clinical relevance remains challenging and requires attention and standardization [37,74]. SNV panels are usually designed to contain known variants, often with known clinical effect. This makes them easy to implement in clinical practice with standardized variant to haplotype translations. Sequencing data, on the other hand, contain more variant information and allow for the extraction of additional variants should they become of interest. Therefore, sequencing based translations can be updated with the development of more guidelines and insights into variant effects. Nonetheless, this does come with more intense data processing.

### 5.2. Imputation

To expand the number of interrogated SNVs, imputation can be used for technologies that do not cover the entire genome (Table 2). Imputation is predominantly used in GWAS analysis and to expand the PGx panel in genome wide arrays [8]. With imputation, the presence of a genomic variant is inferred based on the absence or presence of a linked SNV. These predictions all come with a probability for the occurrence of the SNV of interest. Often only imputations with a high probability are included (e.g., >90%) to avoid inaccurate assignments. Reisberg et al. have shown that imputation accuracies as high as 99% can be reached for PGx variants [8]. Nevertheless, a probability of 90% also means that there is a 10% change that the imputed variant is not correct. While this is certainly acceptable for population studies, it is not sufficient for tailoring drug treatment in an individual patient. Furthermore, to reach high imputation accuracy, an imputation dataset specific to each patient’s ethnical background is needed as the level of linkage disequilibrium (LD) between two SNVs can differ between different populations [8,75]. One clear example of the differences in LD between populations is the HLA tagging SNVs; to identify the HLA-A*3101 allele, associated with carbamazepine toxicity, a linked SNV is used. In Caucasians, the rs1061235 (NC_000006.12:g.29945521A>T) variant is in full LD with the *3101 haplotype, therefore the presence of the HLA-A*3101 allele can be inferred based on the presence of the rs1061235 variant [76]. However, in the Asian population, this variant is not in LD with the *3101 allele. For individuals of Asian descent, the rs1633021 (NC_000006.12:g.29779092T>C) variant can be used as a linked SNV as this variant is in LD with HLA-A*3101 in this population [77]. Using the Caucasian-linked SNV in the wrong population can lead to errors in the inferred haplotype, phenotype and ultimately lead to treatment errors. Therefore, the application of imputation should be limited to research purposes until the reliability for an individual patient has been proven.

### 5.3. Haplotype Phasing

**Figure 1:**
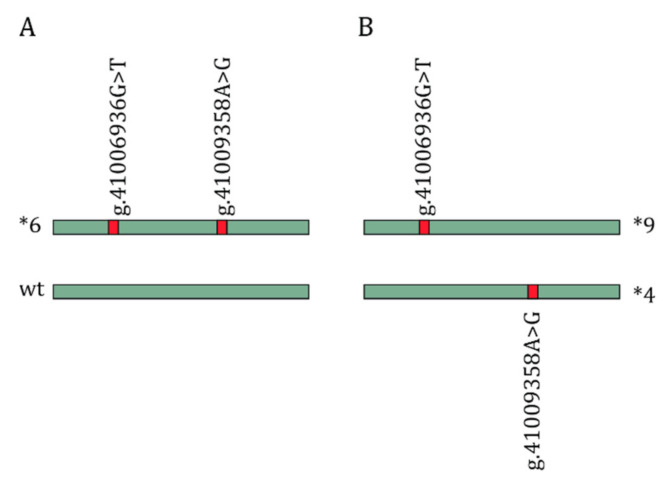
Haplotype phasing in CYP2B6. Inability to phase the rs3745274 (NC_000019.10:g.41006936G>T) and the rs2279343 (NC_000019.10:g.41009358A>G) to the correct allele can result in differences in *-haplotype assignment. Panel (**A**) shows the most common situation in individuals who are heterozygous for both variants, the CYP2B6*1/*6 diplotype. Panel (**B**) shows the alternative conformation where variants are located on opposing alleles leading to a CYP2B6*4/*9 diplotype. Both situations result in the same predicted CYP2B6 metabolizer phenotype (intermediate metabolizer). Conventional methods using linkage disequilibrium assume the variants to be located on the same allele, resulting in a CYP2B6*6 assignment. wt: Wild type.

In addition to variant expansion, imputation can also be used for haplotype phasing (Table 2). With haplotype phasing it can be determined if variants are located on the same allele or if they are on different alleles, potentially leading to differences in phenotype assignment [10,75,78]. The problem of phasing only exists when >1 heterozygous variant is present. However, given the polymorphic nature of many pharmacogenes, the likelihood of identifying multiple heterozygous variants within the gene locus of interest is highly likely [79]. The *-nomenclature is designed to describe only the variants in one allele, assuming that the variants have been phased into two separate alleles. Nonetheless, there are no clear guidelines available describing in what manner variants should be assigned to either one of the alleles. Both CPIC and the DPWG report which diplotypes translate into which phenotypes and occasionally which variants are needed to assign a specific haplotype but not on the phasing of variants. Haplotype phasing can, however, make the difference between a Poor Metabolizer (two loss-of-function variants on different alleles) and an Intermediate Metabolizer (two loss-of-function variants on the same allele and no variants on the other allele). Especially since many pharmacogenomic haplotypes are characterized by multiple variants, in theory all of them can have an impact on protein function. Including phasing in pharmacogenomics can, in some patients, improve haplotype assignments and therefore phenotype prediction. One example is the *CYP2B6* gene for which phasing has been shown to be relevant [10]. When the rs3745274 (NC_000019.10:g.41006936G>T) and the rs2279343 (NC_000019.10:g.41009358A>G) variant are both detected, conventional methods assume they are located on the same allele based on linkage disequilibrium and assign a *6 haplotype (Figure 1). Additionally, CYP2B6*6 is the more common haplotype in most populations. The CYP2B6*6 haplotype occurs around 10% in Asians and up to 40% in the African population. CYP2B6*4 and *9 occur between 0 and 5% in all populations [80]. Clinical data show that in 1.5% of the individuals who carry both these variants, they are located on different alleles, resulting in a *4/*9 haplotype. In this case, both a CYP2B6*1/*6 and a CYP2B6*4/*9 call result in the same phenotype making the clinical impact limited [5]. However, for other variants, this might not be the case. Imputation could be used to infer haplotype phasing, by using the linkage between two observed SNVs to predict if they are located on the same allele or on different alleles. Nevertheless, the same limitations to imputation as described above apply [10].

With NGS, it is possible to phase read to their allele of origin without the need for pedigree information or computational phasing. For NGS, linked reads can be used for this purpose. Linked-read sequencing is based on partitioning the DNA with barcoded gel beads resulting in barcoded short fragments which can then be sequenced with conventional short-read methods. Due to the barcodes, every read can be linked back to the original position and artificial long input DNA can be reconstructed [81]. For long-read sequencing, the length of the reads in itself can be utilized for haplotype phasing. By overlapping the long-reads, large haploblocks can be formed. Nonetheless, large regions which are homozygous can cause an inability to phase large haploblocks [64].

### 5.4. Structural Variants

It has been shown that the majority of pharmacogenes is largely characterized by complex regions, such as CNVs, structural rearrangements and repetitive sections (Table 1) [82]. Full gene deletions or duplications of *CYP2D6* occur in 5–10% of the population [83]. Nonetheless, not all arrays contain probes that can directly detect CNVs. To still be able to obtain CNV data from microarrays, several tools are available. Examples of these types of tools are PennCNV [84], QuantiSNP [85], GenoCN [86] and Nexus [87]. These tools make use of the B allele frequency and the log R ratio which are extracted from the array data. SNP array make use of common SNPs indicated by an A or B (allele) variant. In a normal situation of two copies, there is either an AA, AB or BB at a specific locus. The presence of a deletion or a duplication can be derived from an aberrant number of either an A or a B allele frequency, which is reflected in the B allele frequency parameter. The log R ratio is the log of the ratio between observed and expected intensity values at each variant. It reflects the intensity of the signal at each variant site, a deviation from the expected intensity signal. [88,89]. While results seem promising [8], full validation of these tools for PGx is still needed.

NGS data can resolve all SNVs in the sequenced region, nevertheless, it is difficult to assess CNVs based on sequencing data alone. To aid CNV calling with NGS, several tools have been developed; XHMM [90], CoNIFER [91], Varseq [92] and CNVnator [93], all of which use sequencing depth as an indication of a gene deletion or duplication. These tools do require large datasets and a sufficient range in depth to identify CNVs; details of the use of sequencing depth for CNV calling have been reviewed previously [90,94,95]. Yao et al. tested these three tools on their performance on CNVs of different sizes. Unfortunately, the agreement between the methods was low and there was a bias towards the smaller CNVs as opposed to large CNVs, potentially caused by the limitations of read length [94]. Nonetheless, the advancements in this field evolve rapidly, leading to several laboratories which are now able to reliably identify CNV based on short-read sequencing data. Efforts of using sequencing based CNV calling for *CYP2D6* have shown mixed results [51,56]. Cohn et al. were able to accurately determine CNV status for 87 out of 98 patients. For nine, the CNV calls were inconclusive, and for a further two, there was a discrepant call between sequencing-based CNV calling and a targeted panel [51]. Gulilat et al., including 235 subjects, were able to confirm all sequencing-based CNV variants with panel-based testing [56].

The distinction between a pharmacogene and a pseudogene can be even more challenging. For example, *CYP2D6* and *CYP2D7* share >98% of their sequence, making it difficult to determine from which gene a sequencing read originates [67,96,97]. Due to the relatively short reads (100–200 bp), these complex regions cannot always be well characterized by NGS as reads are not long enough to distinguish between different locations in the complex region [98]. Long-read sequencing, on the other hand, allows for unambiguous mapping of a sequence to the gene of origin without interference of pseudogenes. Additionally, complex regions can be solved in one long read. Indels (deletions or duplications of 1–1000 base pairs) cannot always be accurately determined with short reads as the indel length might surpass the maximum read length. With a read length around 10 kbp in long-read sequencing any structural variants within this maximum length can be covered in one read [64,67,68].

The detection of CNVs in *CYP2D6* is routine in clinical practice. However, the full characterization of the complexity of pharmacogenes is still in the research phase, relying on the further development of long-read sequencing and bioinformatic tools.

### 5.5. Variants of Unknown Effect

A clear benefit of sequencing over SNV panels is the increase in the number of variants that can be identified. While SNV-panel approaches remain limited to the pre-selected variants, sequencing data can help identify variants in the entire sequenced region, including rare variants (Table 2). As mentioned previously, over 90% of the identified variants in pharmacogenes are classified as a rare variant [99,100,101] (Table 1). Additionally, rare variants are expected to be more deleterious than common variants resulting in a potential higher impact on protein function [82]. To collect the most data on rare and novel variants, a WGS or targeted whole gene sequencing approach would be most suitable. Nevertheless, due to a lack of knowledge regarding the impact of these variants, they cannot yet be applied in clinical practice [83,98,101,102,103]. As they are by definition not commonly observed, it is difficult to assign a functional effect. Several strategies have been proposed to detect the impact of rare variants, of which the most common options are the use of cell-line models, in silico predictions or by studying patients displaying the most extreme phenotypes [104]. For clinical application, in vivo studies are most suitable. However, due to the low frequency of these variants, it is nearly impossible to have an appropriate sample size to study these variants [99,105]. In vitro analyses are more easily accessible and generally show a good indication of the effect of a particular variant. Nonetheless, in vitro findings can deviate from the in vivo situation and can still be too laborious for high throughput analyses. Therefore, for high throughput variant predictions, the in silico approach is most desirable. In silico models are based on sequence conservation, the physiochemical and crystal structure of the protein, or on evolutionary scores [102,106]. One, or a combination, of these factors is used to predict the impact the variant will have on enzyme function. To assess the applicability of in silico tools in pharmacogenetics, Han et al. conducted a study to test the accuracy of these tools. They showed that for 10 selected SNPs, the best models accurately predicted the functionality of 80% of the SNPs [107]. In addition, Hao et al. showed that 68% of non-synonymous SNPs in phase II enzyme genes were correctly predicted to be damaging [108]. These results indicate that the applicability of these assays is still limited. Ultimately, collecting more genetic, accompanying clinical data and better prediction models can help us understand the role of these rare variants to be able to use them in clinical practice.

### 5.6. Pharmacogenomics and Disease Genes

Moreover, variants that are disease predictors can be encountered. Complex examples of this are genes which are both pharmacogenes as well as disease-causing genes. *RYR1* is linked to an increased risk of malignant hyperthermia which could classify it as a disease gene and, as such, it is included in the ACMG guidelines [109]. However, one of the factors that could cause the MH in susceptible patients is volatile anesthetics which could classify *RYR1* as a pharmacogene, as well as it interacting with drugs [110].

In summary, the number of available genotyping technologies for PGx has evolved rapidly in recent years and continues to expand. Ultimately, selecting the right technology is not a matter of fact but a matter of choosing the right technique for the right problem.

## Figures and Tables

**Table 1:** Characteristics of pharmacogenes in CPIC and DPWG guidelines. Related drugs are all drugs with dose recommendations. A drug can be related to multiple genes and therefore counted more than once. The locus size and the number of known rare variants (the number of variants with a minor allele frequency < 1% in an aggregated population, including singletons) are extracted from Gnomad (GRCh38). Part of the locus defined as complex is the percentage of the locus defined as a repeat or segmental duplication extracted from UCSC browser (https://genome.ucsc.edu). CPIC: Clinical Pharmacogenetic Implementation Consortium, DPWG: Dutch Pharmacogenetics Working Group.

Protein	Gene	Related Drugs		Locus Size (bp)	Rare Variants, n (% of Known Variants)	Part of Locus Defined as Complex, %(bp)
		CPIC	DPWG			
CACNA1S	*CACNA1S*	7	-	73,055	2520 (98%)	33.3
CFTR	*CFTR*	1	-	250,187	1684 (99%)	42.2
CYP2B6	*CYP2B6*	1	1	27,149	761 (98%)	100.0
CYP2C9	*CYP2C9*	10	2	50,734	632 (98%)	72.0
CYP2C19	*CYP2C19*	15	10	90,525	712 (99%)	83.6
CYP2D6	*CYP2D6*	14	21	4408	992 (97%)	100.0
CYP3A5	*CYP3A5*	1	1	31,833	643 (98%)	49.4
CYP4F2	*CYP4F2*	1	-	20,098	766 (97%)	51.4
DPD	*DPYD*	2	4	917,258	1211 (98%)	40.0
FACT. V LEIDEN	*FACT. V LEIDEN*	-	1 *	72,423	1679 (97%)	41.9
G6PD	*G6PD*	1	-	16,183	465 (98%)	36.4
HLA-A	*HLA-A*	2	1	4625	423 (71%)	100.0
HLA-B	*HLA-B*	6	7	87,698	308 (78%)	62.1
IFNL3	*IFNL3*	2	-	1577	317 (95%)	100.0
IFNL4	*IFNL4*	2	-	3543	404 (97%)	100.0
NUDT15	*NUDT15*	3	3	9656	244 (99%)	64.7
RYR-1	*RYR1*	7	-	153,866	6584 (98%)	51.4
SLCO1B1	*SLCO1B1*	1	2	108,045	951 (96%)	69.6
TPMT	*TPMT*	3	3	26,764	346 (97%)	52.3
UGT1A1	*UGT1A1*	1	1	13,052	470 (99%)	40.3
VKORC1	*VKORC1*	1	3	5139	370 (98%)	41.8

* This interaction is aimed at the entire group of drugs classified as oral contraceptives with estrogen.

**Table 2:** Performance and applicability of available genotyping methods used for PGx. PGx: pharmacogenomics, WES: whole exome sequencing, WGS: whole genome sequencing. NA: not applicable, in this case due to a whole gene/genome coverage and therefore no need for imputation in this region. This table aims to serve as a guide to help select the best technology for the problem at hand. ++ indicates the best score on the parameter, + indicates a good score, - indicates a bad score, -- indicates the worst score on the parameters. Depending on the specific purpose, the weight of the parameters in the selection of the appropriate technology may vary.

		SNV Panel		Short-Read Seq			Long-Read Seq	
		PGx Panel	Whole Genome Panel	PGx Panel	WES	WGS	PGx Panel	WGS
Turnaround Time Wetlab *		++	+	+	+	+/-	-	--
Haplotype phasing	Computational	-	+/-	+	+/-	+	++	++
	Direct	-	-	-	-	-	++	++
Imputation		-	+/-	+/-	+/-	NA	NA	NA
Coverage of PGx variation		+	+/-	++	+/-	++	++	++
Detection of rare variants ^†^		+	+	++	+/-	++	++	++
Detection of variants outside the predefined gene/variant panel		--	--	-/+	-/+	++	++	++
Detection of structural and complex variants		--	--	+	+/-	+	++	++
Turnaround time data processing *		++	++	+	+	+/-	-	--
Costs ^‡^ [29]	Investment	++	+	-	-	-	-	-
	Running costs per sample	+/-	++	+/-	+/-	-	+/-	-

^†^ For SNV panels, it is assumed that the variants are present in the selected variant panel. * A short turnaround time is indicated by the +. ^‡^ Lower costs are indicated by the +.
